# The Status of Data Management Practices Across German Medical Data Integration Centers: Mixed Methods Study

**DOI:** 10.2196/48809

**Published:** 2023-11-08

**Authors:** Kerstin Gierend, Sherry Freiesleben, Dennis Kadioglu, Fabian Siegel, Thomas Ganslandt, Dagmar Waltemath

**Affiliations:** 1 Department of Biomedical Informatics at the Center for Preventive Medicine and Digital Health Medical Faculty Mannheim Heidelberg University Mannheim Germany; 2 Core Unit Data Integration Center and Medical Informatics Laboratory University Medicine Greifswald Greifswald Germany; 3 Institute for Medical Informatics (IMI) Goethe University Frankfurt University Hospital Frankfurt am Main Germany; 4 Department for Information and Communication Technology (DICT), Data Integration Center (DIC) Goethe University Frankfurt University Hospital Frankfurt am Main Germany; 5 Chair of Medical Informatics Friedrich-Alexander-Universität Erlangen-Nürnberg Erlangen Germany

**Keywords:** data management, provenance, traceability, metadata, data integration center, maturity model

## Abstract

**Background:**

In the context of the Medical Informatics Initiative, medical data integration centers (DICs) have implemented complex data flows to transfer routine health care data into research data repositories for secondary use. Data management practices are of importance throughout these processes, and special attention should be given to provenance aspects. Insufficient knowledge can lead to validity risks and reduce the confidence and quality of the processed data. The need to implement maintainable data management practices is undisputed, but there is a great lack of clarity on the status.

**Objective:**

Our study examines the current data management practices throughout the data life cycle within the Medical Informatics in Research and Care in University Medicine (MIRACUM) consortium. We present a framework for the maturity status of data management practices and present recommendations to enable a trustful dissemination and reuse of routine health care data.

**Methods:**

In this mixed methods study, we conducted semistructured interviews with stakeholders from 10 DICs between July and September 2021. We used a self-designed questionnaire that we tailored to the MIRACUM DICs, to collect qualitative and quantitative data. Our study method is compliant with the Good Reporting of a Mixed Methods Study (GRAMMS) checklist.

**Results:**

Our study provides insights into the data management practices at the MIRACUM DICs. We identify several traceability issues that can be partially explained with a lack of contextual information within nonharmonized workflow steps, unclear responsibilities, missing or incomplete data elements, and incomplete information about the computational environment information. Based on the identified shortcomings, we suggest a data management maturity framework to reach more clarity and to help define enhanced data management strategies.

**Conclusions:**

The data management maturity framework supports the production and dissemination of accurate and provenance-enriched data for secondary use. Our work serves as a catalyst for the derivation of an overarching data management strategy, abiding data integrity and provenance characteristics as key factors. We envision that this work will lead to the generation of fairer and maintained health research data of high quality.

## Introduction

**Figure 1:**
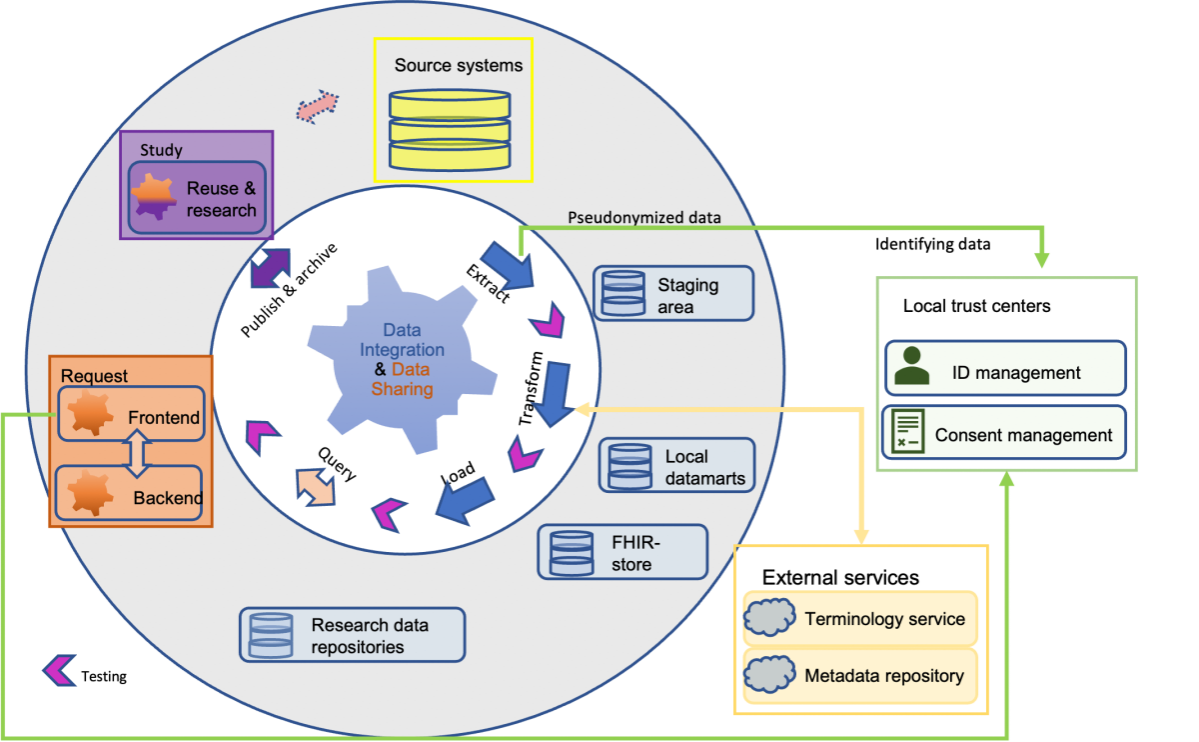
Data life cycle and data management processes. An overview of core processes and artifacts from data management practice in a Medical Informatics in Research and Care in University Medicine data integration center. FHIR: fast health care interoperable resources.

Data integration centers (DICs) within the German Medical Informatics Initiative (MII) have evolved rapidly in the past years [1-4]. DICs process and provide digital medical data for the secondary use in research. The foundation of data sharing (DS) and interoperability within the MII is an agreed-upon common core data set (CDS). The basic modules are generic and include data items encoding laboratory results, diagnosis, procedures, or medication data. The extension modules contain domain-specific data such as oncology or microbiology data [5]. The CDS data items are processed using a standardized extract-transform-load (ETL) development process that follows the data life cycle (Figure 1). Specific testing measures throughout the data processing chain are implemented to ensure accuracy and high quality. The architecture of every Medical Informatics in Research and Care in University Medicine (MIRACUM) DIC (see also Figure 1) is built upon the medical informatics reusable ecosystem of open source linkable and interoperable software tools [6]. Data requests by researchers are limited to and based on generic institutional policies and a defined legal framework. The concrete status of the DICs with respect to enabling the findable, accessible, interoperable, reusable (FAIR) principles still needs to be determined [7]. However, several initiatives have already outlined the importance of applying the FAIR principles for both input and output data [8-10].

The data life cycle describes the journey of biomedical data from data collection to final analysis and publication (Figure 1). Particularly when working with (sensitive) patient data, the understanding of the data’s origin and the relationship between an element and its predecessors, also called traceability (see Textbox 1), is highly relevant for legal requirements and a fundamental prerequisite of data quality. “Black box” processing and reporting of findings based on routine data should no longer be acceptable [11] since it may lead to loss of data and contextual knowledge about the data [12]. This is a reason why the DICs faces an increasing pressure to implement thorough data management concepts, in particular provenance. An option is the adoption of generic provenance concepts from the World Wide Web Consortium (W3C) [13]. However, the application of these concepts requires insights and understanding of the data management tasks in the given context.

Insufficient information about data formation processes and metadata (see Textbox 1) pose validity risks and can impede the quality assessment of extracted clinical data and related processes. Data with unknown provenance and lack of traceability endure from a confidence deficiency and therefore minimize the acceptance for secondary use.

**Related terminologies.**

- Provenance (World Wide Web Consortium [W3C] working definition): “Provenance of a resource is a record that describes entities and processes involved in producing and delivering or otherwise influencing that resource. Provenance provides a critical foundation for assessing authenticity, enabling trust, and allowing reproducibility. Provenance assertions are a form of contextual metadata and can themselves become important records with their own provenance” [13].
- W3C provenance: is a family of specifications for provenance with a generic concept to express specific meta-information (or metadata) about data and its related artifacts. Provenance records contain the agents (eg, people and institutions), entities (eg, data sources and data elements), and activities (eg, extract, load, and transform), involved in producing, influencing, or delivering a piece of data or a thing. The granularity of the W3C provenance concepts influences the level of traceable data management activities [13]. Provenance can be distinguished as data and workflow provenance [14].
- Meta-information (or metadata): machine understandable information for the web [15]. Metadata contain substantial characteristics to express (provenance) information for any kind of artifacts during data managing and play a crucial role in the implementation of the findable, accessible, interoperable, reusable (FAIR) principles [7].
- Traceability: ability to retain the identity of the product and its origin [11]. Traceability is essential to ensure data integrity and trust in the data [16]. In our study traceability is the ability to trace (identify and measure) all the steps that led to a particular point in a data transformation process. Traceability assumes enrichment of data with proper meta-information.

In this work, we seek clarification about the data management processes in German DICs. We aim to facilitate a comprehensive understanding and transparency of these processes to boost data reliability and integrity. We therefore ran a mixed method study across all MIRACUM DICs to get a picture of current traceability and verifiability of patient data and metadata processed from heterogeneous clinical data sources. We expect that DICs would benefit from an increased focus on governance of data management practices rather than random or only partly managed data processing. To support the change, we offer a maturity framework which can be implemented in DICs for self-evaluation. We hypothesize that the framework will foster the implementation of improved data management processes, transparency, traceability, and better provenance tracing.

## Methods

### Study Design

This study uses a mixed methods design [17] and associated best practices [18]. A mixed methods design leads to more plausible and comprehensible quantitative outcomes if combined with qualitative statements. The design involved the collection of qualitative and quantitative data in a single interview and subsequent analysis to strengthen the study’s conclusions. The collection of quantitative and qualitative data was performed concurrently on the same survey and with the same priority. The study has been reported according to the Good Reporting of a Mixed Methods Study (GRAMMS) checklist [19] (Multimedia Appendix 1). Based on the survey results and discussions among the authors, a maturity framework was developed, following the capability maturity model (CMM) [20].

### Study Settings and Participants

The study was performed as a semistructured interview. The interview questions cover clinical data processing and provenance practices within the DICs. The results from a MIRACUM workshop on FAIR data management and discussions with data experts from different DICs contributed to the design of the questionnaires. In addition, we build the questions upon insights from a survey on the research field of provenance [14].

For this work, we distinguished data management operations that concern the data integration (DI) phase (blue items in Figure 1) from operations concerning the DS phase (orange items in Figure 1). Thus, the interview questions were split into 2 separate questionnaires, containing 16 questions (DI) and 38 questions (DS), respectively. The DI questions covered data management activities during the extraction, transformation, and loading of electronic health records. DS questions comprised available documentation of resources, activities for DS processes, and organizational information. The interview does not cover the management of patients’ consent since it is a precondition for data processing and release from the DICs [3].

The questions were numbered and grouped by subject. A mixture of open and closed questions was chosen to get a more comprehensive insight into the respective fields. The questionnaires were created in German language and pilot-tested internally with data experts.

Stakeholders from each MIRACUM site participated in the interview. We provided the questionnaires in advance with the option to delegate the task to accountable staff members. This kept both the interviewer and the participant in line, avoided distractions, and encouraged an open communication. Participants consent was obtained in written form ahead of the actual interview.

### Sample

A total of 10 DICs (all MIRACUM sites) were invited to participate. We subsequently collected data from all sites with 22 participants, thereof 4 women and 18 men contributors. Due to the COVID-19 pandemic, all interviews were conducted virtually. The interviewing person shared the screen with the questionnaire displayed on it while the interview was conducted. Qualitative and quantitative data were collected in German language based on the participant’s answers during the interview phase. All data were concurrently entered into a database (Research Electronic Data Capture [REDCap; Vanderbilt University]) by the interviewing person during the interview [21]. The data collection took between 1.5 hours and 4 hours per DIC. Overall, the data collection period lasted over 3 months. Due to the interview technique no missing data occurred.

### Data Collection

The data collection method relied on asking questions within the predetermined thematic framework. Even for closed questions, there was always the option to ask additional questions and to store the answers.

The data collection included quantitative and qualitative data with equal emphasis (Multimedia Appendix 2). The qualitative data were collected in free-text fields and during the interviews with stakeholder professionals (Multimedia Appendix 2). The data collection took between 1.5 hours and 4 hours per DIC. Before starting the data analysis, all collected data were translated into English and covalidated.

### Data Analysis

After performing the semistructured interview, we conducted a thematic analysis. We converted or transformed qualitative data into quantitative scores or constructs by “coding” the qualitative responses into different groups. We identified common topics or patterns and ensured that these patterns appropriately represent the participants’ responses using the 4-eyes-principle.

The analyses were conducted anonymously without identifying the respective DIC. The tables and figures outline the individual characteristics and frequency counts were calculated. The categorical variables are described using counts and percentages, if applicable. The data were described using median and range for the continuous variables, if applicable. The figures were created with R (version 4.2.0; The R Foundation) [22]. Qualitative, free-text data were read, analyzed, and coded, if necessary. The narratives representing the coded themes were produced from the data material. The data analysis was reviewed by all authors.

### Integration

Qualitative data were combined with quantitative data whenever possible. Thus, the qualitative results were integrated with the corresponding quantitative results and then presented numerically. The outcome was reported as descriptive statistical results. Whenever integration was not possible, we reported qualitative results instead. After analysis of the qualitative and quantitative data, the preliminary findings were presented and discussed among the authors.

### Ethical Considerations

The ethics approval was waived by the University of Heidelberg or Mannheim University Medicine Ethics Committee II. Informed consent was obtained from all subjects (the stakeholders) to participate in the interview about the status of their data processing pipelines. All study data are deidentified. The participants did not receive any compensation.

## Results

### Overview

In our study, we seek clarification about the data management processes in German DICs. We aim to facilitate a comprehensive understanding and transparency of the prevailing practices for data extractions, data transformations, data storage, and data provision to boost data reliability and integrity. We first present the main survey outcomes, and then we introduce a maturity framework.

### Results Overview

All 10 DICs of the MIRACUM consortium participated in the survey between July and October 2021. All 22 participants, either the head of a DIC or a member of the technical staff, responded to a total of 66 questions, thereof 16 questions about the DI phase and 12 questions about the locally used data elements and catalogs from the MII CDS. A total of 9 DICs answered the 38 DS specific question (Table 1); data from the Core Unit Data Integration Center at the University Medicine Greifswald is missing.

**Table 1:** The number of data integration center participants (Medical Informatics in Research and Care in University Medicine) in the 3 survey sections.

	Questions of data integration (n=16), n	Questions of status Medical Informatics Initiative data elements and catalogs (n=12), n	Questions of data sharing (n=38), n
University Medicine Dresden	1	1	1
University Medicine Erlangen	3	3	3
Goethe University Frankfurt	2	2	2
University Hospital Freiburg	1	1	1
University Hospital Giessen	2	2	2
University Medicine Greifswald	4	4	–
University Medicine Magdeburg	2	2	2
University Medicine Mannheim	3	3	3
University Medical Center Mainz	1	1	1
Philipps-University Marburg	3	3	3

### General and Organizational Matters

#### Expectation Regarding Provenance

The interview revealed considerable expectations regarding the collection and use of provenance and metadata information, also beyond the W3C provenance definition (Table 2). Interestingly, the most common expectations were associated with the assessment of data quality (n=7), with traceability and information capability (n=7), and with the transparency in processing steps, workflows, or data sets (n=2). Other frequently named expectations were linked to technical reasons (n=4) such as debugging or performance evaluation. Less frequent terms included compliance with regulations (n=2), reproducibility, support of scientific usage process, or increased confidence in data. Expectations like clear regulation of responsible parties, interoperability, and increased acceptance were mentioned once (n=1). In this, 1 DIC stated no usage of provenance information at all.

**Table 2:** Expectation regarding provenance, a summary of all reported expectations by 10 data integration centers.

	Frequency of expectations regarding provenance, n
Traceability and information capability	7
Data quality assessment	7
Technical reasons	4
Transparency of processing steps	2
Support of scientific process	2
Reproducibility of data flow	2
Proof of compliance	2
Increased confidence	2
Interoperability	1
Internal evaluation about changes in data elements	1
Increased acceptance	1
Clear regulation responsibilities	1
Concurrently no use	1

#### Self-Assessment of Provenance Experience

**Figure 2:**
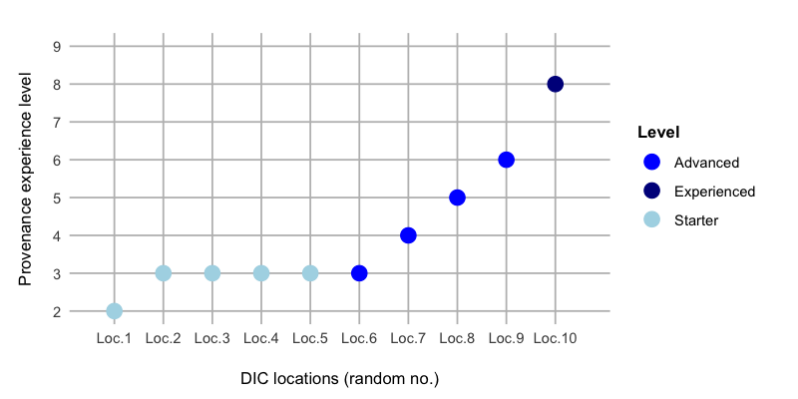
Self-assessment of provenance experience level. All reported self-assessments by the 10 participating data integration centers. DIC: data integration center.

When analyzing the data in Figure 2, we observed a low provenance experience. More than half of the DICs ranked their provenance experience as a starter level with a score 0-3 (n=6). The 3 sites reported an advanced level with a score 4-7. Just 1 site rated their experience with a score of 8 (corresponding to expert level).

#### Organizational Structure

Consistent with the W3C provenance model [13] and Herschel et al [14], the organizational component of a DIC represents a core unit at many German medical faculties. When asked for the organizational prerequisites, all DICs reported that specifications of the manufacturer systems and standard operating procedures (SOPs) were available. However, the degree of maturity varies across DICs.

At the time of the interview, all DICs (n=10, 100%) were in a continuous development process with drafted SOPs at different levels. However, some DICs (n=3) already reported gaps in their SOPs, preventing the full coverage of process flows for DI and DS. Nearly half of the sites (n=4, 40%) used already approved SOPs. Roles and responsibilities, as central parts of the SOP, had been defined in most DICs (n=8, 80%). Only a few DICs (n=3, 30%) had a dedicated role concept (Figure S1 in Multimedia Appendix 3).

### Availability of Metadata and Related Tool Usage

No consequent and targeted practice for provenance capture could be determined. We hypothesize that it might be difficult to develop a standardized, structured, and machine-readable metadata schema across all German university hospitals. Similar results regarding insufficient availability of (semantic) metadata for provenance were observed [23]. Detailed results for the individual questions are given in Figure S2 in Multimedia Appendix 3.

### Metadata Exploitation During Data Management

#### Overview

The development of metadata schemata is an important factor for high traceability [16]. Hence, we were interested in learning how organizational and document resources might help to generate metadata and to embed metadata within the digital object itself. This analysis section targeted the annotation status such as labeling of data elements, data sets, or tagging of files. Detailed results are available in Figures S3-S5 in Multimedia Appendix 3.

#### Documentation Matters

All interviewees declared that data management activities were not subjected to specific data management planning or tools. Any planning or preparational documentation was collectively performed using tools such as JIRA (Atlassian) [24] or Confluence (Atlassian) [25]. During the DS phase, most DICs (n=8) follow internal SOPs for the documentation of methods, or data management plans, respectively. All other DICs reported that internal, project-specific tools were applied. Processes were partially under construction.

#### Documentation Artifacts From Data Elements and Coding in Data Integration Phase

Appreciably all sites (n=10) reported about their level of documentation for accessing the source systems, for the maintenance of the data elements, for code development and execution, as well as the content of log files as part of their ETL-process as described below.

#### Annotation of Data Elements

As expected, and in line with the literature [26], preliminary attempts for data annotation exist. However, these attempts do not yet cover the whole processing pipeline in all DICs. The applied annotation approaches vary, too. It is noteworthy that the best, and partially automatic annotation was yielded on the joint segment Fast Healthcare Interoperability Resources (FHIR) to the research data repository (RDR; n=10), since this pipeline is part of the MIRACUM standard ETL process [3]. Detailed results are available in Figure S3 in Multimedia Appendix 3.

#### Log Files for Improved Traceability

Log files are text-based files, which include timestamps, store events, processes, and transactions. Thus, log files provide valuable provenance information. However, a direct access to log files was not possible during this study due to the risk of disclosing critical or sensitive information.

Most DICs (n=9) already established log files to trace environment and execution information, particularly during DI. In most cases, the log files contain configurable parameters and elements, mostly generated within the respective infrastructure framework. Some frameworks comprised self-defined information and messages for error, warning, and execution statistics. Depending on the actual process, short- or long-term retention could be observed. Long-term retention was applied for data transfer logs and short-term for application logs, for example, throughout the ETL life cycle. Half of the DICs (n=5) reported that the access to source systems is automatically logged with user information and time stamps, but without relationship to the particular data elements. In general, access to the source-application itself is not possible. More than two-thirds of the centers (n=4) have manual logging features in place. Only 1 DIC does not perform any logging (n=1).

Only sparse information was provided about the computational environment and execution workflows during runtime of scripts in productive operation (Figure S4 in Multimedia Appendix 3). A small number of sites (n=2) reported that automated and collaboratively accessible information were created. The 3 centers (n=3) said that no such information was generated. All other survey participants (up to n=7) asserted that the logging protocols were either compiled manually or generated automatically. Based on the survey data no systematic approach was deducible.

However, half of the DICs reported that scripts which are executed during the data requesting phase often do not produce log files. If log files were produced by the scripts, they contained information about execution and error history (n=4). Many DICs emphasized their capability of access-logging to data pools, computational environments, and execution history. The recorded information includes details about Docker containers such as the software status of the environment. However, some data seems to be missing in the logs, including the date of execution or the user account. Logs from the RDR Informatics for Integrating Biology and the Bedside indicated access logins (who and when) and querying of data elements. Extraction protocols were produced for some source systems.

#### Versioning Information Status

Version information, an important element for reproducible research, creates a history for each file. Based on the annotation of the source code and artifacts, for example, the used programming language, provenance data can create relationships between individual elements or documents. Figure S5 in Multimedia Appendix 3 illustrates that the generated code was in general subjected to textual documentation, and code was mostly versioned in a DI pipeline (n=10). GitHub is mainly used as version control tool. Importantly, the DI segment FHIR to the RDR provided full versioning capability in all DICs (n=10). The reason is that MIRACUM developed and delivered a centralized component for this workflow step [3]. Also, code for the ETL segment for data processing from staging servers to a FHIR repository is highly version controlled. Lower coverage was observed on the initial stretch source system to staging (n=7). In this, 1 interviewee explained that this circumstance was due the code being in the responsibility of the manufacturer. Another expert said that code was managed manually (n=1). In 1 case, no version control was implemented at all. The situation is similar when data is queried by scripts for research purposes since code versioning was tool-guided by the most DICs (n=7). Overall, the results suggest that version information is available, but needs to be prepared in more detail to be useful for provenance processing.

#### Documentation Artifacts of Testing Procedures and Script Validation

**Figure 3:**
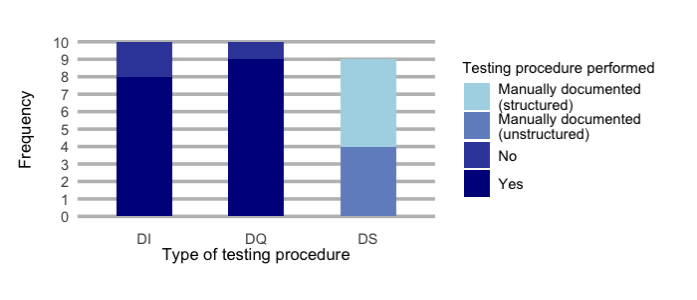
Testing or validation procedures. A summary of all reported types of testing procedures during the scripting phase. Data integration centers reporting about their testing procedures. DI: data integration phase; DS: data sharing phase; DQ: data querying phase.

A considerably high number of DICs confirmed the implementation of test procedures (n=8) and data quality measures (n=9; Figure 3).

Notably, different test documentation strategies were reported by the stakeholders (Figure S6 in Multimedia Appendix 3). Most sites (n=4) mentioned the provision of an automated testing documentation which was collaboratively accessible for the authorized staff during the data integrity measurements pipeline.

Data quality is mainly assessed using the Data Quality Assessment reporting tool, which has been developed within MIRACUM (n=6) [27]. A total of 2 DICs used self-developed assessment and documentation of data quality.

All DICs validate their data querying scripts. Evidence for validation is provided by manually documenting the queries in a structured and permanently accessible way on GitLab (GitLab Inc) [28] or JIRA [24] (n=5). Unstructured evidence such as the four-eyes principle was practiced in 4 DICs.

#### Documentation Artifacts From Final Review and Facts About Research Result Objects

All previous processes and individual outcomes contribute to the history of the so-called result object. We anticipate that the result object should contain all provenance-related metadata. As shown in Figure S7 in Multimedia Appendix 3, most participants (n=5) examine all the documentation and artifacts for traceability. Applied examination methods included the 4-eyes-principle, random sample checks, or ETL checklists with defined examination criteria. Approximately one-third of the respondents (n=3) indicated that the traceability of documentation and related artifacts was not checked. Only 1 DIC has plans to check traceability systematically and automated. Remarkably, examination of the result object regarding adherence to FAIR principles and provenance assessment was not performed in any DIC. These findings indicate a lack of awareness for FAIR data management, as has also been observed in a recent survey within the German Network University Medicine [29].

### Derivation of a Maturity Framework

**Figure 4:**
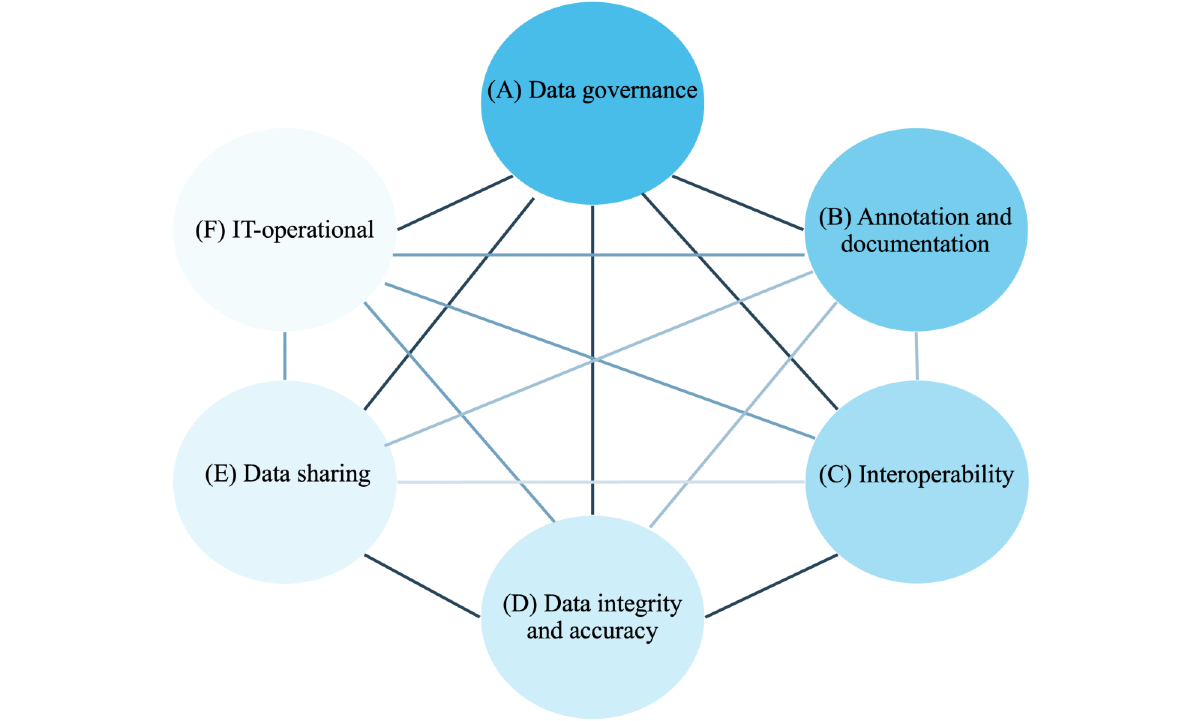
Dimensions and their relationships within the framework for provenance tracking.

On the basis of our study results, we derived a data integration center toward maturity framework (DIC2MF), which incorporates the specific needs and metadata items of German DICs (Figure 4). The DIC2MF indicates a DIC’s readiness status for provenance tracking (“provenance power”) and can be used as a benchmarking tool.

### Dimensions and Categories of the Framework

The DIC2MF concept is based on the CMM. Unlike the already published maturity model for provenance management, which was established in the hydro- and geoscientists’ field [30], our approach comprises 6 dimensions and related categories (Figure 4) which together constitute provenance characteristics that a DIC requires to be effective in delivering traceable and reliable patient data for secondary use. The dimensions and categories were influenced by the grouping of key interview findings from (1) related organizational, legal, and technical conditions, (2) the metadata exploitation based on data annotation and documentation degree and associated operations, and (3) including the measures to ensure quality during the different operations. Textbox 2 elucidates the proposed framework and the associated characteristics.

**Figure 5:**
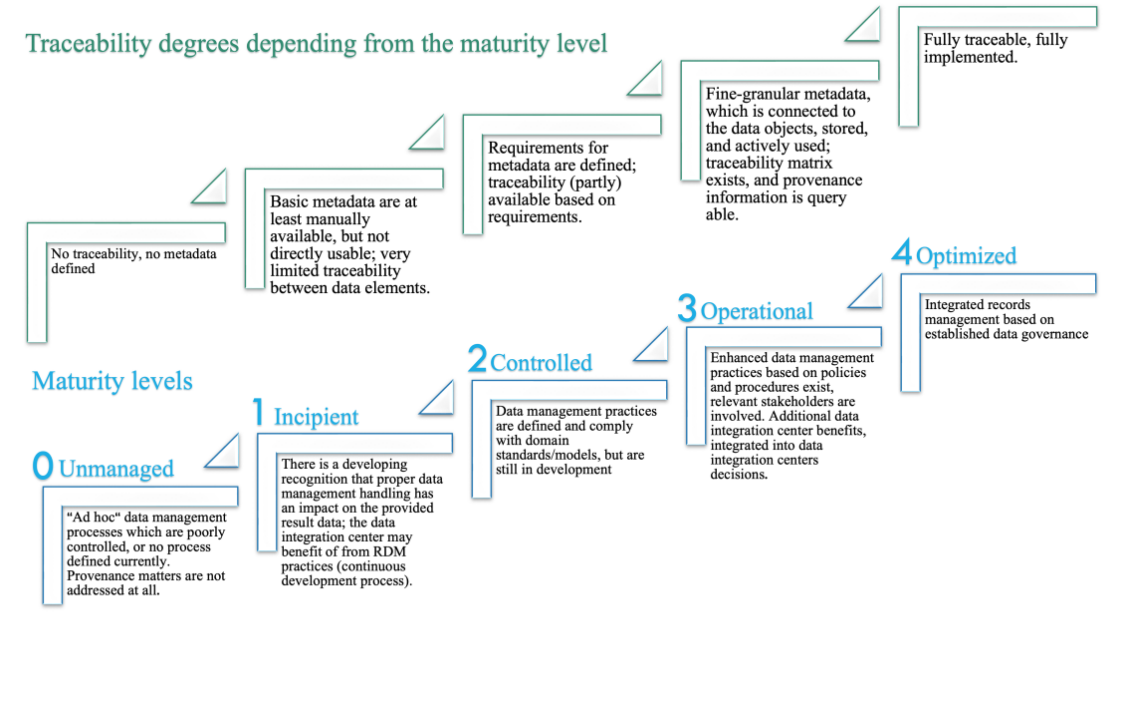
The 5 maturity levels in the framework (data integration center toward maturity framework) and defined degrees of traceability.

**Figure 6:**
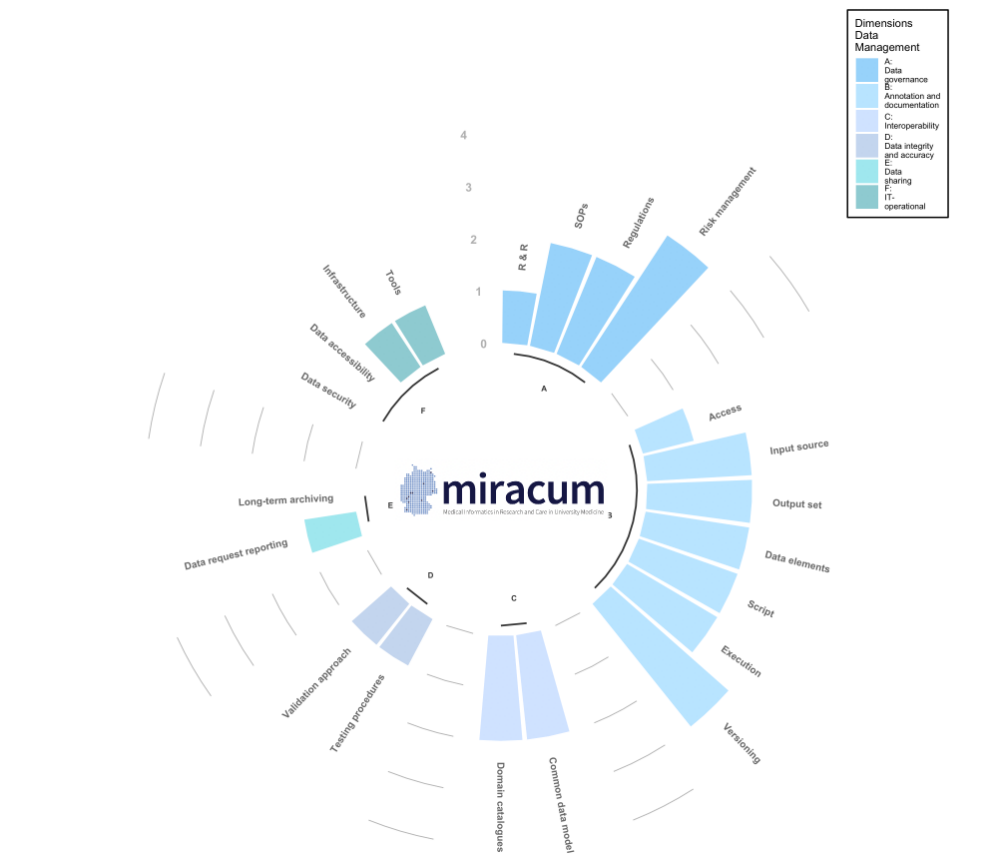
DIC2MF—provenance power as part of the data management maturity framework. The DIC2MF indicates the DIC’s readiness status for provenance (“provenance power”). Logo used with permission from the Medical Informatics in Research and Care in University Medicine (MIRACUM) Consortium [2]. DIC2MF: data integration center toward maturity framework; SOP: standard operating procedure.

Each dimension is represented by a specific ability level. Figure 5 depicts the different gradations of the 5 ability levels “unmanaged,” “incipient,” “controlled,” “operational,” and “optimized.” Each level describes a degree of traceability fulfillment and is an indicator for the provenance power in the DIC. The completeness and quality of traceability goes hand in hand with the levels of maturity. An instantiation of the framework is shown in Figure 6.

**Components of the framework for provenance tracking (data integration center toward maturity framework).**

- Data management dimensions and categories
  - (A) Implement “Data governance” which explores the availability of important legislation, guidelines, or rules that directly relate to the scope of a data integration center
    - Roles and responsibilities (staff, roles, and training)
    - Standard operating procedures (quality management)
    - Regulations (eg, general data protection regulation and patient consent)
    - Risk management (controlling risks)
  - Build multiple data management dimensions (B up to E) for data processing and data analysis
    - (B) Addresses the practices to “Annotation and Documentation” of data and the related processes
      - Considers metadata about the management of the (automated) documentation and annotation steps of the individual data and process elements, including the provenance of any processed element
        - Access
        - Input sources
        - Output sets
        - Data elements
        - Scripts
        - Execution
        - Versioning
      - Considers information from log files created during data conversion, for example, to cover the facets of provenance according to the World Wide Web Consortium provenance recommendation (see Textbox 1)
    - (C) Enforces the transformation and processing of data into interoperable formats to enable translational research with patient data
      - Includes metadata about the usage of standard data models and catalogs
        - Common data model
        - Domain specific catalogs
    - (D) Examines the implementation of quality standards to ensure “Data Integrity and Accuracy” of the processed patient data
      - Comprises metadata about all methods for examining and maintaining the data quality
        - Testing procedures
        - Validation approach
    - (E) Data sharing
      - Includes metadata about the service of organization and reporting of the data request and analysis result as well as taking care of long-term archiving aspects
        - Organization and reporting
        - Long-term archiving
    - (F) IT-operational
      - Comprises metadata about
        - Data security of patient data
        - Data accessibility of patient data
        - Infrastructure and computation environment
      - Tools and software
- Relationship
  - It should be mentioned that relationships exist between the dimensions, for example, data processing must adhere to given data governance rules

### Instantiation of the Framework

The inner circle (Figure 6) represents the grouped 6 data management dimensions for provenance tracking (following the specification in Figure 4). Each dimension contains multiple categories and each category reflects the substantial characteristics for the expression of provenance. The quality of provenance expression can be derived from the ability scale (between 0 and 4) which defines how reliably and maintainably the implemented practices within a DIC can produce the required outcomes. The higher the bar the more provenance information is available. Thus, the height of the bar is an indicator for the need to improve data management practices given on the description of the ability level. For example, progress from 1 maturity level to the next one may be reached by adding fine-granular metadata in compliance with the W3C provenance components agent, activity, and entity in a second step. The presented concepts are a first step toward identifying the requirements for traceability within a DIC.

## Discussion

### Principal Findings

We successfully performed a mixed method study and gained deep insight into the status of data management processes in the German medical DICs. Our work facilitates understanding and traceability and will potentially boost the reliability and integrity of data for secondary use. We derived a maturity framework and applied it as a benchmark to measure the degree of traceability and deriving from this the provenance power of individual data elements in MIRACUM German DICs. The proposed maturity framework for provenance readiness helps DICs to identify their conceptual bottlenecks in provenance tracking and increases trustful dissemination of clinical data.

We hypothesize that our work could serve as a catalyst for an overarching data management strategy for DICs. The beneficial approach presented here could be implemented widely as a common assessment tool, within the MII structure and in the medical research field itself.

### Evaluation

#### Framework Applicability

The framework can be used for critical systematic self-evaluation. It can guide the identification of relevant components for provenance tracking and thus facilitate traceability of patient’s data processing. The information obtained from the framework dimensions A to F help to develop the necessary metadata, and consequently enhance traceability on process and element level.

#### Establishing Traceability and Best Practices

Establishing traceability is one of the biggest challenges associated with any data conversion. A combination of several aspects may lead to the condition that traceability has not been implemented effectively at the DICs. Predominantly, a lack of awareness and provenance expertise could be a key finding from the self-assessment of provenance experience (Figure 2) and indicates a subordinate role of provenance to date. A lack of technological framework may furthermore hinder the uptake of provenance in the data processing pipelines. Here, the traceability issue can be linked to a lack of granularity including details about workflow steps and about the processed data elements themselves. ETL pipelines are mostly implemented individually by the DICs. Practices in the highly ranked centers for provenance expertise revealed that these include annotation and metadata documentation, even if it is not always machine-readable and automatically recorded.

A tentative explanation is that there is no systematic approach for gathering provenance data of individual data items (Table 2). The procedure of tracking data set or data items is neither formalized nor sufficiently standardized. Consequently, no targeted provenance collection and metadata concept has been established as of now. In addition, sparsely developed traceability decreases the reliability and thus the quality of single data elements for secondary use (Figure 3). Even if general testing procedures are available in the DI pipelines, there is a lack in quality traceability.

The following examples showcase how DICs may increase their maturity level by using the proposed framework dimensions and categories while connecting metadata to the associated artifact: (1) dimension A foresees (a) guidance on data managing activities, like define operations by SOPs, introduce data management plans, and consider legal restrictions and (b) regular data management training for the responsible staff. Connect both topics at least on data and process level. (2) The challenge of dimension B could be passed step by step (a) while gaining and deriving targeted annotation from log files for building and filling the maturity framework on a data element level, log files are configurable and enable the traceable storage of events so that these can be analyzed and optimized. In this way, log files thus help to track data and their processes, and to reconstruct transactions. Elements of log files could be selected as in the proposed framework, for example, source and target system, information about type of event or logged action, version or actor; (b) by having appropriate, clear, and complete documentation for all measured data in place, if possible, in machine-actionable way and connect this information to the data; (c) by making metadata accessible and adding richer prospective and retrospective provenance metadata. These actions will allow for fine-grained versioning workflows linking to outputs produced during the distinct executions of ETL pipelines. The metadata approach should consider information derived from the W3C components agents (such as developer and data owner), activities (such as different programming scripts), entities (such as data sources or data elements). (3) Convert the extracted data into common and interoperable health care standards as defined in dimension C and connect the associated metadata information to your processed data as described in dimension B. (4) Testing and validation (dimension D) approaches add quality information to the processed data itself. Collect available metadata on applied activities to ensure data quality as given in dimension B. (5) Dimension E, dedicated to the DS phase enriches a data element with information from the data requesting, reporting, and archiving phase. (6) Dimension F intends to collect meta-information about the operational environment in which the data were collected and processed.

### Related Work

Provenance tracking and granularity issues were addressed in different papers [31,32]. Gierend et al [33] performed a scoping review on provenance in biomedical research and offered comprehensive results concerning the practical application of provenance and the associated challenges, including aspects like completeness and validation and provenance granularity issues. Curcin et al [34] reported that both data and processes need provenance, gathered in consistent, interoperable manner to make research results verifiable and reproducible. These works directed our study approach to examine the traceability aspect. Johns et al [35] tried to figure out knowledge on provenance methods in a more general way. Regarding the term provenance, Herschel et al [14] pointed to the definition of provenance, which leaves room for many different interpretations of and approaches to provenance and investigated the question why capturing provenance is useful. This led us to clearly define the goal of our study and give clear expectations regarding provenance accomplishment. Furthermore, this might give clear expectations regarding provenance accomplishment and provide the framework for the scope and the extent of implementation measures. In the same way, the outcome of our study can be used by the recently launched community-driven project which aims to define a “MInimal Requirements for Automated Provenance Information Enrichment” guideline [36]. The projects’ goal is to build a general data model and semantics for provenance in the biomedical community.

Training issues were addressed as a challenge of poor data management practice [26]. Better health informatics training and permanent data manager and software architect positions are demanded in health research groups. This indicates that our maturity framework needs an iterative and interdisciplinary approach to implement traceability in data processing pipelines.

### Lessons Learnt

During the conduction of the semistructured interview and the implementation of our framework, we learnt that the extent of the complex processing steps requires interdisciplinary team work to come to a proper level of provenance granularity. We are convinced that the community will benefit from a consequent exchange with stakeholders from different areas of expertise, like medical experts, data owners, and computer scientist. In addition, we encountered a major increase of transparency and traceability since we started with a consequent application of the maturity framework approach in our DIC. Moreover, having data governance in place, would facilitate the FAIR oriented data management planning and as such boost the data asset to be more reliable and trustful for or in the research field. Another recommendation is to spend more time on training in this field.

### Ongoing Processes

Changing conditions in clinical routine, in granularity of requirements (decision-making, identifier management, and legal matters) demand continuous adaptation of the framework. We foresee extensions for provenance representation and storage, provenance retrieval, and usability along discussion for risk and benefit.

There are recent advancements to transform the dimension and categories into the W3C provenance concepts. We introduce a first provenance implementation in our DIC in Mannheim (University Medicine Mannheim DIC) in a proof-of-concept study in peer review phase.

### Limitations

Our investigation is limited to the MIRACUM DICs, to their current service profiles and development stages as well as to the experience of the involved staff. Since provenance data are sporadically available, we were not able to consider maintainability aspects of provenance. Derivation of qualitative and quantitative results to the framework levels was performed by means of an evaluative description of metadata availability and the ability of traceable data. Integration of pseudonymization and consent management are external processes and not in primary scope for this study.

### Conclusions

Implementing traceable data life cycles and transparent data management processes are sophisticated and challenging tasks, not only for the MIRACUM DICs. Notwithstanding, sufficient traceability would enable data to be a trusted asset in the medical DIC. Our paper provides insights on how institutions (attempt to) implement data management principles to provide clinical routine data for secondary use. However, to implement traceability, explainability of the relationships and the order between the data and process elements are required. We discussed the extensive transformations, curations, and linked artifacts of collected data elements and workflows during the entire data life cycle. The obtained insights led us to identify possible improvements and actions. One such action is the introduction of a maturity framework which visualizes the specific traceability challenges on a technical and organizational level observed at each DIC. In future, we seek to derive a generic provenance model and common data provenance strategy based on the traceability findings. To this end, we will investigate how complete provenance, as part of a FAIR data management strategy, can be delivered and what the limitations are in this regard. We envision that this work will lead to FAIR and maintained health and research data of high quality.

## Appendix

Multimedia Appendix 1 Good reporting of a mixed methods study checklist [19].

Multimedia Appendix 2 Data collection items (in English and German language).

Multimedia Appendix 3 Additional results from the survey.

## Abbreviations

CDS: core data set
CMM: capability maturity model
DI: data integration
DIC: data integration center
DIC2MF: data integration center toward maturity framework
DS: data sharing
ETL: extract-transform-load
FAIR: findable, accessible, interoperable, reusable
FHIR: Fast Healthcare Interoperability Resources
GRAMMS: Good Reporting of a Mixed Methods Study
MII: Medical Informatics Initiative
MIRACUM: Medical Informatics in Research and Care in University Medicine
RDR: Research Data Repository
REDCap: Research Electronic Data Capture
SOP: standard operating procedure
W3C: World Wide Web Consortium
