# Supplementary material for: The Status of Data Management Practices Across German Medical Data Integration Centers: Mixed Methods Study
Source: J Med Internet Res. 2023 Nov 8;25:e48809. doi: 10.2196/48809 (PMC10666010; doi:10.2196/48809)
Supplement: Multimedia Appendix 1 [file jmir_v25i1e48809_app1.pdf]

Good Reporting of A Mixed Methods Study (GRAMMS) checklist [19]

Title: The Status of Data Management Practices Across German Medical Data Integration Centers: Mixed-Methods Study

**Good reporting of a mixed-methods study (GRAMMS) guidance**	**Guidance met? (Section: page number in the manuscript)**
Describe the justification for using a mixed methods approach to the research question	Yes<br>- Introduction and Methods/Study Design: pages 2-3<br>- Limitations and Methodological Implications: page 14
Describe the design in terms of the purpose, priority and sequence of methods	Yes<br>- Methods / Study Design: page 3
Describe each method in terms of sampling, data collection and analysis	Yes<br>- Methods:<br>Study settings and participants: pages 3<br>Sample: page 3<br>Data collection: page 4<br>Data analysis: page 4
Describe where integration has occurred, how it has occurred and who has participated in it	Yes<br>- Methods/Integration: page 4
Describe any limitation of one method associated with the present of the other method	Yes<br>- Discussion/Limitations and Methodological Implications: pages 12-14
Describe any insights gained from mixing or integrating methods	Yes<br>- Discussion: pages 12-14
