# Supplementary material for: The Status of Data Management Practices Across German Medical Data Integration Centers: Mixed Methods Study
Source: J Med Internet Res. 2023 Nov 8;25:e48809. doi: 10.2196/48809 (PMC10666010; doi:10.2196/48809)
Supplement: Multimedia Appendix 2 [file jmir_v25i1e48809_app2.pdf]

Title: The Status of Data Management Practices: a Mixed-Method Study across German Medical Data Integration Centers

Overview Data Collection Items (English language) and original questionnaires (German language)

Overview Data Collection Items

Quanti-tative	general items	- participating Miracum Sites<br>- general items related to DIC regulations, SOPs and associated responsibilities<br>- processes description and division<br>- provenance, self assessment, expectation, tools in use, collection
	items in data integration process	- artifacts and their properties from data management activities during data extraction and data transformation phase regarding<br>- data elements<br>- versioning<br>- logfiles<br>- execution history and environment<br>- testing procedures during data management activities<br>- traceability examination of data management activities<br>- existence of metadata concept<br>- usage of metadata template and/or tools
	items in data sharing process	- availability of documentation of resources and activities for data sharing process<br>- availability of organizational information related to data release process: researcher, ethics committee, UAC, data protection officer, consent of data owner, accepted data use agreement, data delivery<br>- items related to querying the data pool based on the data request in order to deliver a result object<br>- generation of querying script<br>- documentation of script available<br>- log files available<br>- content of log files<br>- logging of access to data pools<br>- logging of information about execution environment and history<br>- validation available<br>- format of delivery of result object<br>- storage of data request artifacts<br>- items related to the result object and data delivery<br>- compliance given to the usage regulations<br>- traceability examination of result object and artifacts

		- performed<br>- internal registration of result object and artifacts performed<br>- access medium to result object for researcher<br>- acknowledgement of receipt obtained from researcher<br>- archiving of result object performed<br>- evaluation of result object for compliance to FAIR criteria<br>- metadata concept available for data sharing process
	data interoperability via using terminology services:<br>data element groups from MII core data set and their catalogs status from source system until FHIR profile	- diagnosis, procedure, laboratory finding, medication, intensive care parameter (physiological measurements and therapeutic measures), biospecimens<br>- used coding catalogs and version per data element group and status of FHIR mapping for selected data elements<br>- additional need for metadata
Quali-tative	list of comment fields:	- description to obligatory demands for DIC<br>- description DI processes data integration/data sharing<br>- provenance tools in use<br>- defined provenance criteria collected<br>- expectation on provenance<br>- Data Integration process<br>- code properties (versioning) and retention period<br>- logfiles (execution/error protocol)<br>- metadata tools/templates<br>- workflow tools<br>- transparency/traceability aspect revealed in DIC<br>- measurement of data integrity and data quality<br>- content of logs<br>- information automatically collected about IT infrastructure and execution history<br>- FDR repositories used<br>- data generated on execution history and execution environment<br>- Data Sharing process<br>- infos to data sharing process<br>- validation information<br>- content of log-files<br>- formats produced for researchers<br>- permanent availability of script<br>- result object for researcher

		- storage of data request artefacts<br>- compliance given to the usage regulations<br>- internal registration of result object and artefacts performed<br>- access medium to result object for researcher<br>- metadata concept<br>- Status data elements/catalogues<br>- sources of not structured data<br>- status of annotation of data elements

Original questionnaires (German language)

Prozess Datenintegration

Adding new Participant ID **1**

Participant ID 1

Prozess Datenintegration - Allgemeine Fragen

Ihr Standort

* must provide value

- Dresden
- Erlangen
- Frankfurt
- Freiburg
- Gießen
- Greifswald
- Magdeburg
- Mainz
- Mannheim
- Marburg

reset

Welche Vorgaben sind für die Tätigkeiten in Ihrem Datenintegratioszentrum bindend?

* must provide value

- DIZ Satzung
- Nutzungsordnung
- Datenschutzvorgabe, bitte spezifizieren
- Research Data Policy
- Standard-Arbeitsanweisungen (SOPs)
- Vorgaben der Herstellersysteme
- Weitere, bitte spezifizieren

In welche groben Verarbeitungsschritte (Strecken) ist Ihre Datenintegrationspipeline unterteilt?

Erläuterung:

Bitte geben Sie an, in welche elementaren Teilabschnitte der Prozess der Datenextraktion aus den Quellsystemen bis hin zum Erreichen des FHIR Standards und der Überprüfung in ein Forschungsdatenrepositorium untergliedert ist, wie z.B. Quellsystem - Staging - DWH - FHIR - Forschungsdatenrepositorium.

* must provide value

- Strecken wie beispielhaft vorgegeben
  - streaming
  - klassisch per ETL

reset

- andere, bitte spezifizieren

Expand

reset

Sind für die einzelnen Verarbeitungsschritte Standardarbeitungsanweisungen (SOPs) verfügbar?

* must provide value

- nein
- SOPs sind in Entwicklung
- SOPs sind freigegeben
- unbekannt

Sind für die einzelnen Verarbeitungsschritte Rollen und Verantwortlichkeiten definiert?

(Beispiele für Rollen sind z.B. Entwickler, Tester, Administrator, ...)

* must provide value

- nein
- ja, bitte Rollen angeben

reset

Wie schätzen Sie die Expertise Ihres DIZ zur Erstellung und Verarbeitung von Provenance-Informationen während des Datenintegrationsprozesses ein.

* must provide value

Starter Level | Fortgeschrittenes Level | Experten Level

Change the slider above to set a response

reset

Welche Provenance-Tools haben Sie bereits im Einsatz?

* must provide value

- keine Tools
- folgende Tools, bitte nennen

reset

Welche Provenance-Informationen werden im DIZ erhoben?

* must provide value

- bislang keine
- bitte Erhebungskriterien nennen

reset

Welche Erwartung haben Sie an die Verwendung von Provenance-Informationen?

Expand

Datenmanagement während der Datenextraktions- und Datentransformationsphase

Wie dokumentieren Sie Ihre Datenmanagement-Aktivitäten?

**In der Planungsphase**	
☐ keine Dokumentation<br>☐ Dokumentation über einen Datenmanagementplan/-tool<br>☐ anders, bitte spezifizieren	

**In der Ausführungsphase: Datenextraktion aus den unterschiedlichen Quell- Systemen**		
**Zugang zu den jeweiligen Quellsystemen**		
**Die Datenelemente sind ...**	○ nicht dokumentiert<br>○ manuell annotiert (z.B. in Confluence)<br>○ automatisiert annotiert<br>○ sonstig dokumentiert, bitte spezifizieren<br>reset	
**Der Code ...**	**... ist versioniert**	○ nein<br>○ ja, manuell<br>○ ja, über ein Versionierungstool<br>○ sonstiges<br>reset
	**... generiert Log-Files**	○ nein<br>○ ja, statisch<br>○ ja, parametrisierbar<br>reset
	**... ist inhaltlich dokumentiert**	○ nein<br>○ ja<br>reset
**Die Log-Files ...**	**... werden wie lange aufbewahrt?**	○ keine Aufbewahrung<br>○ bitte angeben, wie lange<br>reset
	**... bestehen getrennt aus Ausführungs-Logs und Fehlerprotokoll-Logs?**	○ ja<br>○ andere Aufteilung, bitte angeben<br>reset
**Daten zur Ausführunghistorie/-umgebung des Codes werden generiert für ...**	**... den Testbetrieb.**	○ nein<br>○ ja und manuell dokumentiert<br>○ ja, automatisiert und sind kollaborativ im Zugriff<br>○ sonstiges, bitte spezifizieren<br>reset
	**... den Produktivbetrieb**	○ nein<br>○ ja und manuell dokumentiert<br>○ ja, automatisiert und sind kollaborativ im Zugriff<br>○ sonstiges, bitte spezifizieren<br>reset

In der Ausführungsphase: Datentransformation in FHIR Standard		
**Die Datenelemente sind ...**		nicht dokumentiert<br>manuell annotiert (z.B. in Confluence)<br>automatisiert annotiert<br>sonstig dokumentiert, bitte spezifizieren
**Daten zum Ursprung der einzelnen Datenelemente sind ...**		nicht verfügbar<br>verfügbar zusammen mit dem Datenelement<br>reset
**Der Code ...**	**... ist versioniert**	nein<br>ja, manuell<br>ja, über ein Versionierungstool<br>sonstiges<br>reset
	**... generiert Log-Files**	nein<br>ja, statisch<br>ja, parametrisierbar<br>reset
	**... ist inhaltlich dokumentiert**	nein<br>ja<br>reset
**Die Log-Files ...**	**... werden wie lange aufbewahrt?**	keine Aufbewahrung<br>bitte angeben, wie lange<br>reset
	**... bestehen getrennt aus Ausführungs-Logs und Fehlerprotokoll-Logs?**	ja<br>andere Aufteilung, bitte angeben<br>reset
**Daten zur Ausführunghistorie/-umgebung werden generiert für**	**... den Testbetrieb**	nein<br>ja und manuell dokumentiert<br>ja, automatisiert und sind kollaborativ im Zugriff<br>sonstiges, bitte spezifizieren<br>reset
	**... den Produktivbetrieb**	nein<br>ja und manuell dokumentiert<br>ja, automatisiert und sind kollaborativ im Zugriff<br>sonstiges, bitte spezifizieren<br>reset

In der Ausführungsphase: Datenintegration in Forschungsdatenrepositorium (FDR)

- ☐ i2b2
- ☐ OMOP
- ☐ andere, bitte angeben

**Wie erfolgt die Befüllung des FDRs**	☐ nicht dokumentiert<br>☐ manuell annotiert (z.B. in Confluence)<br>☐ automatisiert annotiert<br>☐ sonstig dokumentiert, bitte spezifizieren	
**Daten zum Ursprung der einzelnen Datenelemente sind...**	○ nicht verfügbar<br>○ verfügbar zusammen mit dem Datenelement<br>reset	
**Der Code ...**	**... ist versioniert**	○ nein<br>○ ja, manuell<br>○ ja, über ein Versionierungstool<br>○ sonstiges<br>reset
	**... generiert Log-Files**	○ nein<br>○ ja, statisch<br>○ ja, parametrisierbar<br>reset
	**... ist inhaltlich dokumentiert**	○ nein<br>○ ja<br>reset
**Die Log-Files ...**	**... werden wie lange aufbewahrt?**	○ keine Aufbewahrung<br>○ bitte angeben, wie lange<br>reset
	**... bestehen getrennt aus Ausführungs-Logs und Fehlerprotokoll-Logs?**	○ ja<br>○ andere Aufteilung, bitte angeben<br>reset
**Daten zur Ausführunghistorie/-umgebung werden generiert für ...**	**... den Testbetrieb**	○ nein<br>○ ja und manuell dokumentiert<br>○ ja, automatisiert und sind kollaborativ im Zugriff<br>○ sonstiges, bitte spezifizieren<br>reset
	**... den Produktivbetrieb**	○ nein<br>○ ja und manuell dokumentiert<br>○ ja, automatisiert und sind kollaborativ im Zugriff<br>○ sonstiges, bitte spezifizieren<br>reset

Welche Informationen werden in die Log-Files geschrieben?

Sind diese strukuriert oder unstrukturiert?

* must provide value

Expand

Welche Informationen werden über die IT Infrastruktur bzw. über die Ausführungshistorie automatisiert aufgenommen?

* must provide value

Expand

Datenmanagement - Testverfahren

Sind Testverfahren während des Datenmanagementprozesses implementiert ...

**... zur Aufrechterhaltung der Datenintegrität?**	◯ nein<br>◯ ja<br>reset	
	**Die Testergebnisse sind ...**	◯ nicht dokumentiert<br>◯ manuell dokumentiert<br>◯ automatisiert abgleitet und kollaborativ im Zugriff<br>◯ anders dokumentiert, bitte spezifizieren<br>reset
**... zur Messung der Datenqualität?**	◯ nein<br>◯ ja<br>reset	
	**Die Testergebnisse sind ...**	◯ nicht dokumentiert<br>◯ manuell dokumentiert<br>◯ automatisiert abgleitet und kollaborativ im Zugriff<br>◯ anders dokumentiert, bitte spezifizieren<br>reset

Wird ein Workflow-Tool benutzt?
* must provide value

◯ nein
◯ ja, bitte spezifizieren
reset

Werden die vorhandene Dokumentation sowie deren Artefakte auf Nachvollziehbarkeit im DIZ hin untersucht?
* must provide value

◯ nein
◯ ja, bitte Methode und Ergebnisse erläutern
◯ ist geplant, bitte spezifizieren
reset

Gibt es ein Metadatenkonzept?
* must provide value

◯ nein
◯ ja, bitte erläutern oder hochladen
reset

Expand

Werden Metadaten Templates benutzt?
* must provide value

◯ nein
◯ ja, bitte spezifizieren welche
reset

Werden Metadaten Tools benutzt?
* must provide value

◯ nein
◯ ja, bitte spezifizieren welche
reset

Prozess Data Sharing - Allgemeine Fragen zum DIZ

Ihr Standort
* must provide value

- Dresden
- Erlangen
- Frankfurt
- Freiburg
- Gießen
- Greifswald
- Magdeburg
- Mainz
- Mannheim
- Marburg

reset

Welche Vorgaben sind für die Tätigkeiten in Ihrem Datenintegrationszentrum bindend?
* must provide value

- DIZ Satzung
- Nutzungsordnung, bitte spezifizieren
- Datenschutzvorgabe, bitte spezifizieren
- Research Data Policy
- Standard-Arbeitsanweisungen (SOPs)
- Weitere, bitte spezifizieren

In welche groben Verarbeitungsschritte ist Ihr Data Sharing Prozesses unterteilt?
z.B. Anfrage- & Vertrag - Datenanalyse - Datenbereitstellung - Archivierung
* must provide value

- wie beispielhaft vorgegeben
- andere, bitte spezifizieren

reset

Einteilung der Verarbeitungs-/Prozessschritte

Expand

Sind für den Data Sharing Prozess SOPs (Standard-Arbeitsanweisungen) verfügbar?
* must provide value

- nein
- SOPs sind in Entwicklung
- SOPs sind freigegeben
- unbekannt

reset

Bitte listen Sie die dazugehörigen SOPs auf:

Sind für die einzelnen Data Sharing Prozesse/Schritte Rollen und Verantwortlichkeiten definiert?
* must provide value

- nein
- ja, bitte angeben

reset

Expand

Wie schätzen Sie die Erfahrung Ihres DIZ zur Erstellung und Verarbeitung von Provenance-Informationen während der Data Sharing Phase ein?
* must provide value

Starter Level | Fortgeschrittenes Level | Experten Level

Change the slider above to set a response

reset

Welche Provenance-Tools haben Sie bereits im Einsatz?
* must provide value

- keine Tools
- folgende Tools, bitte nennen

reset

Welche Provenance-Informationen werden im DIZ erhoben?
* must provide value

- bislang keine
- bitte Erhebungskriterien nennen

reset

Expand

Welche Erwartung haben Sie an das Sammeln und die Verwendung von Provenance-Informationen?
* must provide value

Expand

Datenmanagement während des Prozesses Data Sharing

Wie dokumentieren Sie Ihre Datenmanagement-Aktivitäten?

* must provide value

- keine Dokumentation
- Dokumentation über einen Datenmanagementplan/-tool
- gemäß SOPs
- anders, bitte spezifizieren

reset

Wieviel Zeit verwenden Sie für die Dokumentation der Datenmanagement-Aktivitäten pro Studie bzw. pro Anfrage?

* must provide value

- Zeit nicht erfasst
- weniger als 8 Stunden
- zwischen 8 und 16 Stunden
- mehr als 16 Stunden
- anders, bitte spezifizieren
- nicht zutreffend

reset

In welcher Form sind (Meta-) Informationen über die Machbarkeits- oder Datenanfrage vorhanden?

Erläuterung:

Dazu gehören z.B. die Einschlusskriterien, Name der Anfragenden/Forscher, Datenmanager/Data Steward, Archivierungsstelle, etc.

* must provide value

- nicht strukturiert
- strukturiert
- über ein Template/Tool strukturiert
- strukturiert und maschinenlesbar

reset

Expand

Welche organisatorischen Informationen sind über den Datenfreigabeprozess verfügbar?

	nicht verfügbar	intern verfügbar, dokumentiert	intern verfügbar auf zentraler kollaborativer Platform	nicht Teil des Prozesses, bitte spezifizieren	
**Anfragende/Forschende**					reset
**Freigabe EK**					reset
**Freigabe UAC**					reset
**Freigabe DSB**					reset
**Zustimmung der jeweiligen Data Owner**					reset
**Nutzungseinschränkungen**					reset
**Akzeptierte Datennutzungsvereinbarung/-vertrag**					reset
**Datenausleitung (Datum)**					reset

Bitte listen Sie hier weitere Information zum Freigabeprozess:

* must provide value

Expand

Abfragen im Datenpool basierend auf der Datenanfrage

Wird das Abfragescript validiert?

* must provide value

- nein oder nicht dokumentiert
- manuell dokumentiert (unstrukturiert)
- manuell dokumentiert (strukturiert)
- automatisiert getestet und kollaborativ

falls validiert, bitte angeben wie validiert:

z.B. gemäß Vier-Augen-Prinzip

Expand

Ist das Abfragescript inhaltlich dokumentiert?

* must provide value

- nein
- ja, teilweise nachvollziehbar dokumentiert
- ja, komplett nachvollziehbar dokumentiert

reset

Ist das Abfragescript versioniert?

* must provide value

- nein
- ja, manuell
- ja, über ein Versionierungstool

reset

Erzeugt das Abfragescript Logfiles?

* must provide value

- nein
- ja, als Ausführungs-Log
- ja, als Fehlerprotokoll-Log

Welche Informationen werden in Log-Files geschrieben?

* must provide value

Expand

Werden die Zugriffe auf die unterschiedlichen Datenpools geloggt?

z.B. über einen Audit-Trail

* must provide value

- nein
- ja, bitte erfasste Elemente angeben

reset

Expand

Werden Daten zur Ausführungsumgebung und -historie protokolliert?

* must provide value

- nein
- ja, manuell dokumentiert
- ja, automatisiert und kollaborativ im Zugriff, bitte spezifizieren
- sonstiges, bitte spezifizieren

reset

Expand

Welche Dateiformate werden für die Datenausgabephase erzeugt?

* must provide value

- überwiegend proprietäre Formate, bitte spezifizieren
- offene Formate und maschinenlesbar, bitte spezifizieren
- beides, bitte spezifizieren
- anders, bitte spezfizieren

Expand

Bleiben alle Datensets, die über das Abfragescript hinweg transformiert werden, erhalten?

* must provide value

- nein
- verfügbar auf Anfrage (Zugriffsbeschränkung)
- intern zentral verfügbar ohne Metadaten
- intern zentral verfügbar mit Metadaten
- anders, bitte spezifizieren (auch Teilschritte der Aufbewahrung)

reset

Weitere Information zur Erhaltung von Datensets

Expand

Ergebnisobjekte

Unter Ergebnisobjekt ist der erzeugte Datensatz zwecks Datenausleitung zu verstehen.

Wird das Ergebnisobjekt vor der Ausgabe an den Forschenden auf Einhaltung der Nutzungsordnung nochmals geprüft und das Ergebnis dokumentiert?

- nein
- ja, bitte Elemente der Prüfung beschreiben

reset

Expand

Wird das Ergebnisobjekt sowie dessen Artefakte auf Nachvollziehbarkeit im DIZ hin untersucht?

* must provide value

- nein
- stichprobenweise, bitte erläutern
- ist geplant, bitte spezifizieren

reset

Expand

Wie werden das Ergebnisobjekt und dessen Artefakte intern registriert?

* must provide value

- DIZ intern ohne Dokumentation
- DIZ intern mit Dokumentation
- DIZ intern kollaborativ mit Dokumentation
- offen und permanent verfügbar
- anders, bitte spezifizieren

reset

Wie bzw. über welches Medium erhält der Forschende Zugriff auf die Ergebnisse (digitalen Objekte)?

* must provide value

- eMail
- USB-Stick
- internes Laufwerk
- Plattform, bitte spezifizieren
- andere, bitte spezifizieren

reset

Wird eine Bestätigung vom Forschenden zum Erhalt der angefragten Daten eingefordert?

* must provide value

- nein
- ja

reset

reset

Werden das Ergebnisobjekt und dessen Artefakte archiviert?

* must provide value

- nein
- ja, bitte Metadaten oder Standard spezifizieren

reset

Standard für die Auswahl von Metadaten bei der Archivierung:

* must provide value

Expand

Inwieweit liegen Ergebnisse für die Evaluierung der digitalen Objekte auf die Erfüllung der FAIR-Prinzipien vor?

* must provide value

- keine Untersuchung bzw. nicht dokumentiert
- stichprobenweise, bitte Erfüllungsgrad beschreiben
- komplette Evaluierung, bitte Erfüllungsgrad beschreiben

reset

Expand

Gibt es ein Metadatenkonzept?

* must provide value

- nein
- ja, bitte erläutern oder bitte hochladen

reset

Expand

Bitte stellen Sie weitere (Meta-) Informationen oder das Metakonzept zur Verfügung:

Upload file

Teilstatus Datenintegration

Adding new Participant ID **1**

Participant ID 1

Ihr Standort
* must provide value

- ○ Dresden
- ○ Erlangen
- ○ Frankfurt
- ○ Freiburg
- ○ Gießen
- ○ Greifswald
- ○ Magdeburg
- ○ Mainz
- ○ Mannheim
- ○ Marburg

reset

Welche Daten werden prozessiert?
Mehrfachnennungen sind möglich.
* must provide value

- ☐ strukturierte Daten (z.B. aus Tabellen)
- ☐ halb-strukturierte Daten (z.B. XML Files)
- ☐ unstrukturierte Daten (z.B. Text, Bilder, Mails)

Aus welchen Datenquellen stammen die unstrukturierten Daten?
* must provide value

Expand

Sind die Datenelemente der jeweiligen Module über die gesamte Integrationspipeline annotiert?

Die Annotierung von Datenelementen bedeutet, dass die vorhandenen Variablen eines Datensatzes beschrieben sind in ihrer Struktur und Bedeutung.
* must provide value

- ○ überhaupt nicht annotiert
- ○ teilweise annotiert
- ○ komplett annotiert

reset

In welcher Form liegt die Annotierung von Datenelementen vor?

Mehrfachnennungen sind möglich.

Bitte in nächstem Feld erläutern.
* must provide value

- ☐ nicht vorhanden
- ☐ reine textuelle Beschreibung (z.B. Confluence)
- ☐ maschinen-lesbar und verarbeitbar (z.B. ODM)
- ☐ anders, bitte unten erläutern

Bitte nennen Sie Details zur Umsetzung der Annotierung:

Expand

Matrix - Vorhandensein von Kodierungskatalogen
Bitte geben Sie an, welche Kodierungen und Kataloge in welchen Systemabschnitten vorliegen.

	Quellsystem	Lokales Data Mart	FHIR-Profil
**Diagnose**	☐	☐	☐
**Prozedur**	☐	☐	☐
**Laborbefund**	☐	☐	☐
**Medikation**	☐	☐	☐
**Intensivmedizin (Physiolog. Messwerte)**	☐	☐	☐
**Intensivmedizin (Therap. Massnahmen)**	☐	☐	☐
**Bioproben**	☐	☐	☐

Kodierungskatalog Diagnose
* must provide value

- ☐ ICD
- ☐ SNOMED CT
- ☐ Alpha-ID
- ☐ Orpha
- ☐ sonstige bitte spezifizieren

Kodierungskatalog Prozeduren
* must provide value

- ☐ OPS
- ☐ sonstige bitte spezifizieren

Kodierungskatalog Laborbefund und Artefakte
* must provide value

- ☐ LOINC
- ☐ UCUM
- ☐ sonstige bitte spezifizieren

Kodierungskatalog Medikation und Artefakte
* must provide value

- ☐ ATC
- ☐ ASK
- ☐ sonstige bitte spezifizieren

Kodierungskatalog Intensivmedizin (physiolog. Messwerte)
bitte Datenelement(e) und Katalog mit Version angeben
* must provide value

Expand

Kodierungskatalog Intensivmedizin (therap. Maßnahmen)
bitte Datenelement(e) und Katalog mit Version angeben
* must provide value

Expand

Kodierungskataloge Bioproben und Artefakte
* must provide value

- ☐ SNOMED CT
- ☐ ICD-O
- ☐ sonstige bitte spezifizieren

Version der Kataloge
