# Supplementary material for: The Status of Data Management Practices Across German Medical Data Integration Centers: Mixed Methods Study
Source: J Med Internet Res. 2023 Nov 8;25:e48809. doi: 10.2196/48809 (PMC10666010; doi:10.2196/48809)
Supplement: Multimedia Appendix 3 [file jmir_v25i1e48809_app3.pdf]

Title: The Status of Data Management Practices: a Mixed-Method Study across German Medical Data Integration Centers

This additional file 4 contains the seven additional figures as referenced in the result section: SOP process status (Figure S1), Availability of metadata and related tool usage (Figure S2), Annotation status of data elements (Figure S3), Logging: recording of environment and execution information (Figure S4), Versioning practice (Figure S5), Level of test documentation availability in data management processes (Figure S6), Review of result object (Figure S7)

Add. Figure S1: SOPs process status

Our participant's rating of the SOP process status for the data integration and the data sharing phase

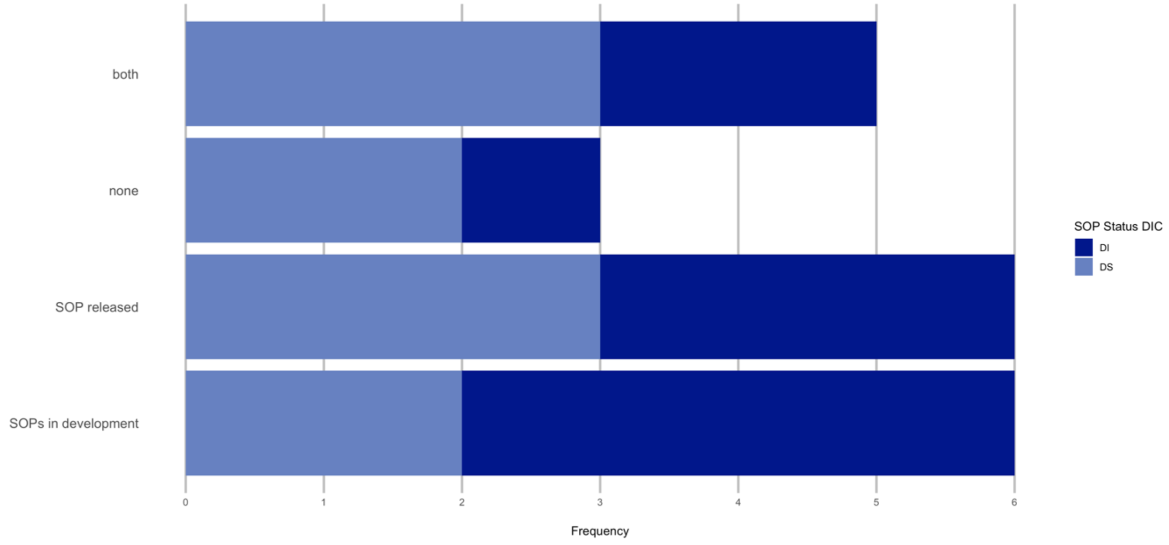

Add. Figure S2: Availability of metadata and related tool usage

Our participant's rating metadata and related tools usage in the data integration (DI) and the data sharing (DS) phase

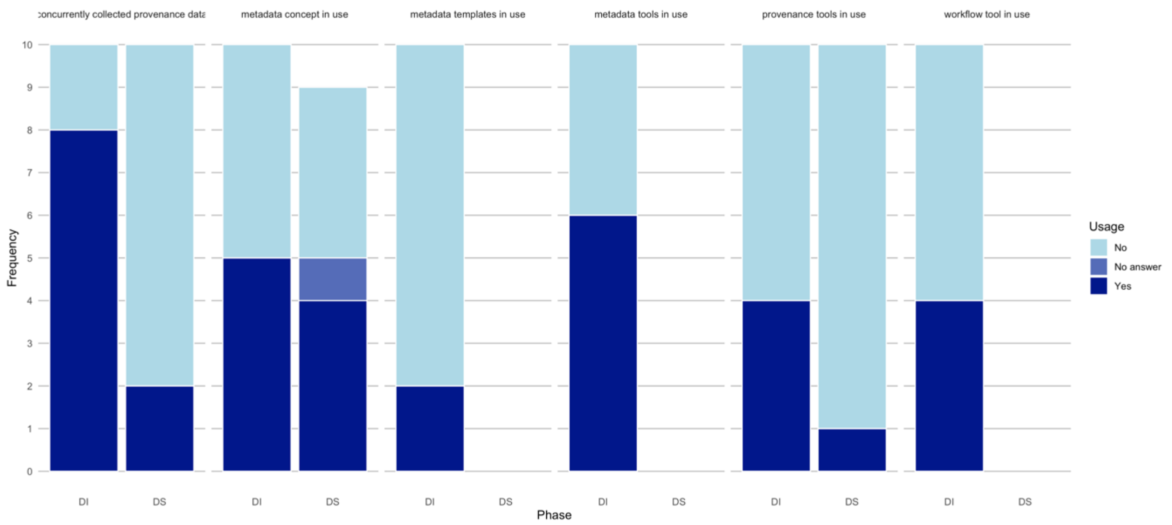

Concurrently collected provenance data. Most DIC (n=8, 80%) declared that they use provenance supporting tools in the data integration phase. In this context, the majority of the DIC involved derived preliminary minimal provenance information. Just under one-third of centers stated that tracking a data set would not yet be formalized. More than half of provenance using DIC indicated to obtain at least the details about the origin of the source system and two thereof mentioned to accomplish FHIR-resource. Two of the seven DIC pointed out that they already processed specific metadata at the file and job level, but not on data element level.

Metadata concept. Exactly half of the participating centers (n=5, 50%) expressed the application or at least initial consideration of developing a metadata concept. One center mentioned to use the MIRACUM cross MDR, another pointed to the FHIR specific metadata, and one more referenced the planned delivery of metadata by the source-system provider and to a general tracking of the tables at all. One center provided no further specification.

Metadata tools. In total, 6 participating centers (n=6, 50%) expressed the application of metadata tools but without having any further metadata implementation in place. The employment of mentioned metadata tools. The MIRACUM MDR, Centraxx-MDR as well as the i2b2 metadata repository were encountered.

Workflow tools. In total, almost half of all DIC (n=4, 40%) stated the usage of workflow tools, whereas all other DIC (n=6, 60%) indicated not to use a workflow tool. The participants mentioned the tools Airflow, GitLab Scheduler, a self-developed tool, automatically derived from streaming practice.

Add. Figure S3: Annotation of data elements.

Participants reported about the annotation status in the data integration pipeline.

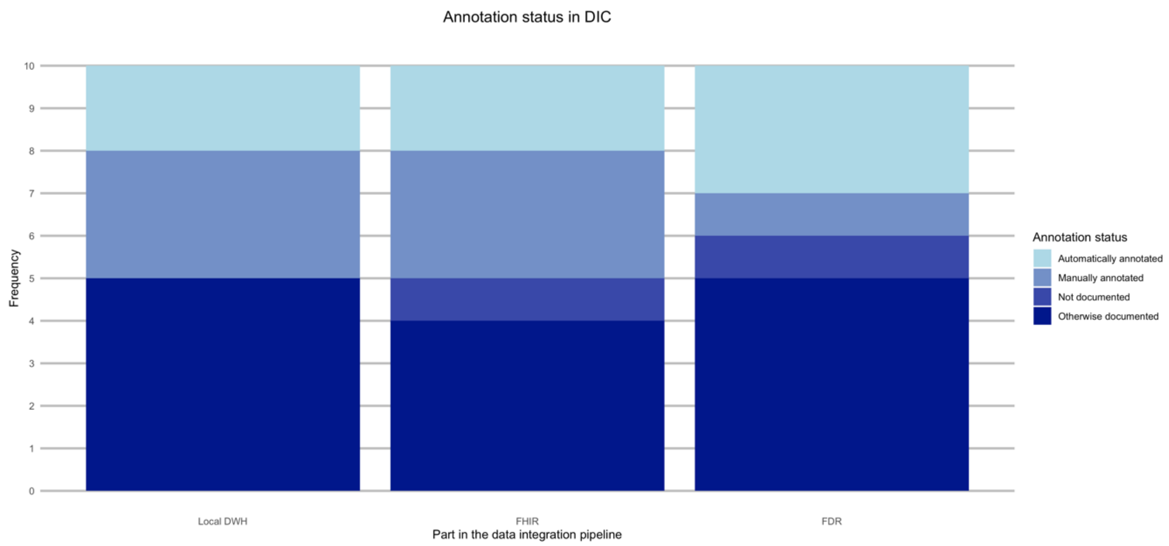

During the first derivation step in the local data warehouse, data elements were mostly manually (n=3, 30%) annotated in collaboration portal (eg., Atlassian Confluence) or 'otherwise documented' (n=5, 50%) while using a metadata repository like Centraxx or the MIRACUM Meta Data Repository (MDR). In the fewest cases (n=2, 20%), automated annotation within the script was reported via the establishment of mapping files.

During the second transformation step into the FHIR structure, one DIC affirmed automated annotation of data elements (n=2, 20%) through scripts, another narrated not having implemented any annotation (n=1, 10%). Manually annotation was performed by three DIC (n=3, 30%). Most DIC (n=5, 50%) stated 'otherwise documented'. As such, some source systems provided directly FHIR resources, or the mapping was held in the MDR.

During the last derivation, from FHIR into the RDR, most DIC (n=5, 50%) mentioned 'otherwise documented', three DIC (n=3) stated 'automated annotation' and one center each (n=1, 10%) said to annotate manually respectively did not document at all.

Add. Figure S4: Logging: recording of environment and execution information
DIC reported about their recording activities regarding computational environment and execution workflow.

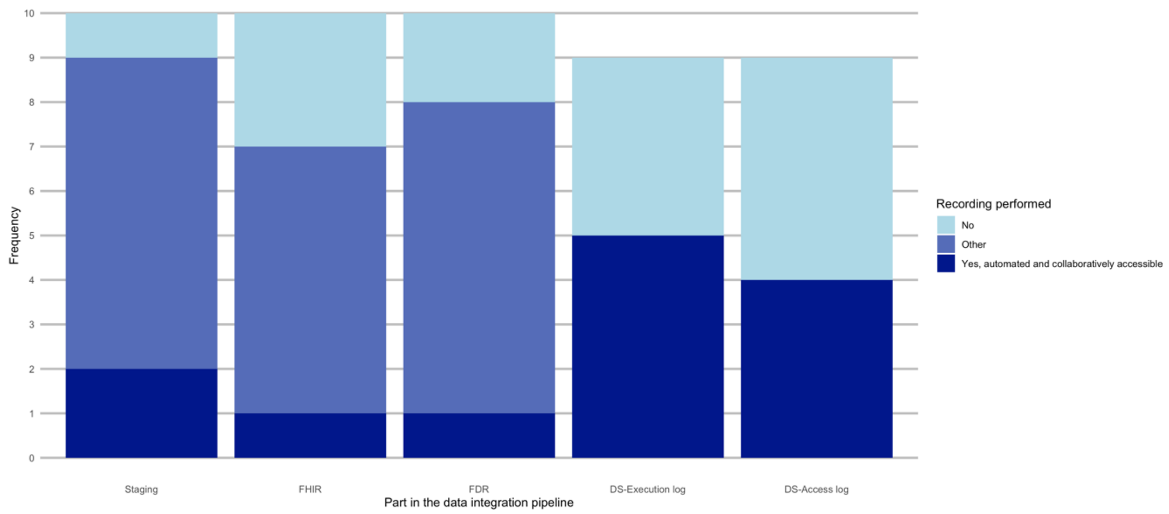

Add. Figure S5: Versioning practice in DIC
DIC reported about their practice in code versioning.

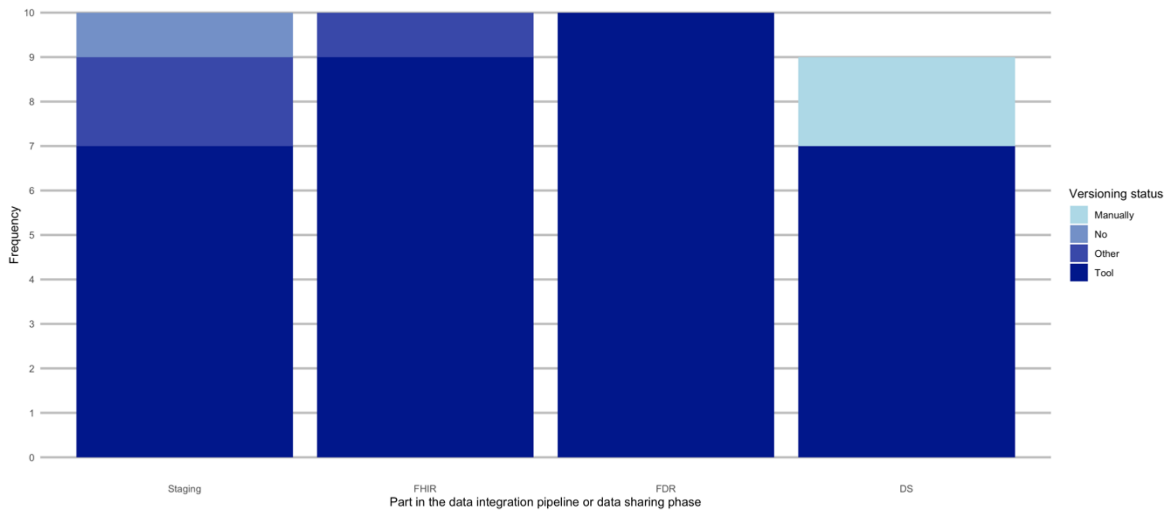

Add. Figure S6: Level of test documentation availability in data management processes
Artifacts from testing procedures and script validation
Participants reported about the test documentation availability.

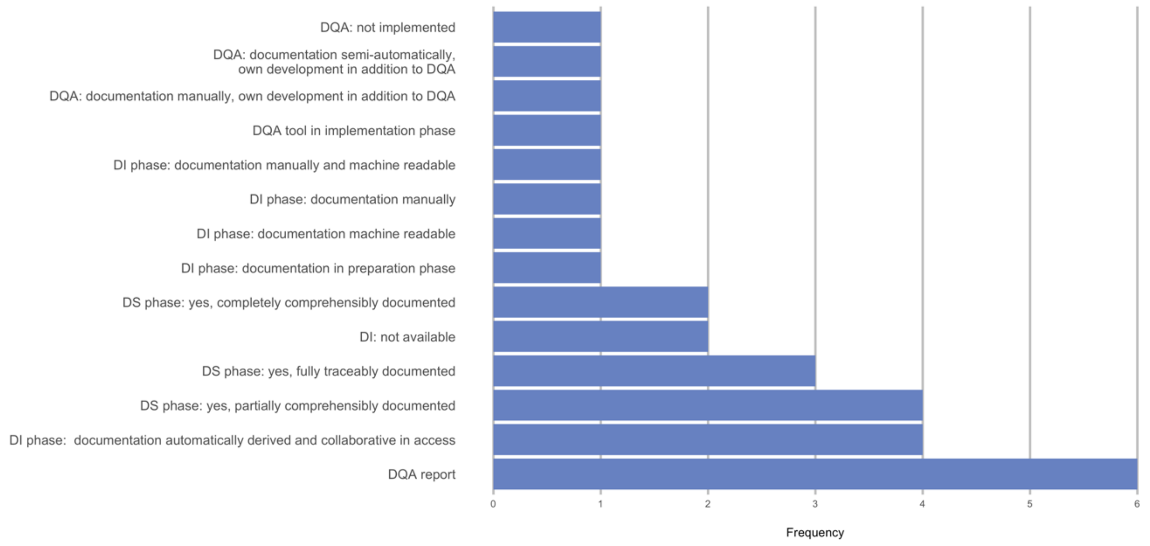

Add. Figure S7: Review of result object
Documentation artifacts from final review and facts about produced research result object.
Participants reported about the transparency in the review process.

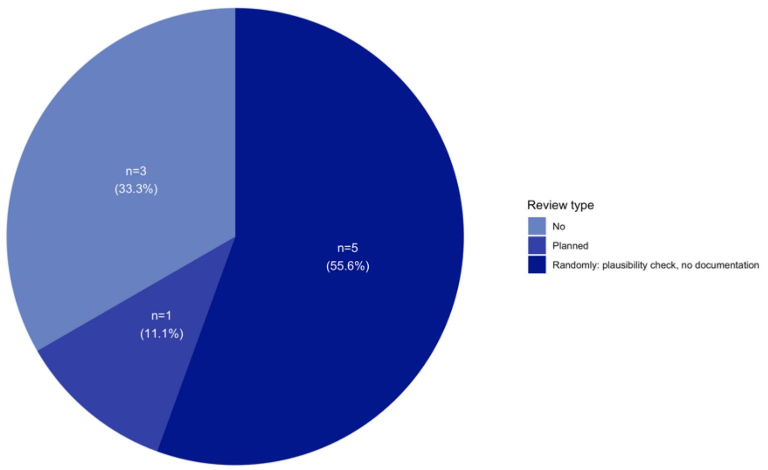
n=3
(33.3%)
n=1
(11.1%)
n=5
(55.6%)
Review type
No
Planned
Randomly: plausibility check, no documentation
